# Propranolol induced G0/G1/S phase arrest and apoptosis in melanoma cells via AKT/MAPK pathway

**DOI:** 10.18632/oncotarget.11599

**Published:** 2016-08-25

**Authors:** Chengfang Zhou, Xiang Chen, Weiqi Zeng, Cong Peng, Gang Huang, Xian'an Li, Zhengxiao Ouyang, Yi Luo, Xuezheng Xu, Biaobo Xu, Weili Wang, Ruohui He, Xu Zhang, Liyang Zhang, Jie Liu, Todd C. Knepper, Yijing He, Howard L. McLeod

**Affiliations:** ^1^ Department of Clinical Pharmacology, XiangYa Hospital, Institute of Clinical Pharmacology, Central South University, Hunan Key Laboratory of Pharmacogenetics, Changsha, China; ^2^ Moffitt Cancer Center, DeBartolo Family Personalized Medicine Institute, Tampa, FL, USA; ^3^ Department of Dermatology, XiangYa Hospital, Central South University, Changsha, China; ^4^ Department of Orthopedics, Hunan Tumor Hospital, Changsha, China; ^5^ Department of Neurosurgery, Xiang-Ya Hospital, Central South University, Changsha, China

**Keywords:** melanoma, propranolol, apoptosis, AKT pathway, MAPK pathway

## Abstract

Both preclinical and epidemiology studies associate β-adrenoceptors-blockers (β-blockers) with activity against melanoma. However, the underlying mechanism is still unclear, especially in acral melanoma. In this study, we explored the effect of propranolol, a non-selective β-blocker, on the A375 melanoma cell line, two primary acral melanoma cell lines (P-3, P-6) and mice xenografts. Cell viability assay demonstrated that 50μM-400μM of propranolol inhibited viability in a concentration and time dependent manner with an IC50 ranging from 65.33μM to 148.60μM for 24h −72h treatment, but propranolol (less than 200μM) had no effect on HaCaT cell line. Western blots showed 100μM propranolol significantly reduced the expression of Bcl-2 while increasing the expressions of Bax, cytochrome c, cleaved capase-9 and cleaved caspase-3, and down-regulated the levels of p-AKT, p-BRAF, p-MEK1/2 and p-ERK1/2 in melanoma cells, after a 24h incubation. The *in vivo* data confirmed the isolation results. Mice received daily ip. administration of propranolol at the dose of 2 mg/kg for 3 weeks and the control group was treated with the same volume of saline. The mean tumor volume at day 21 in A375 xenografts was 82.33 ± 3.75mm^3^*vs.* 2044.67 ± 54.57mm^3^ for the propranolol-treated mice and the control group, respectively, and 31.66 ± 4.67 mm^3^ *vs.* 1074.67 ± 32.17 mm^3^ for the P-3 xenografts. Propranolol also reduced Ki67, inhibited phosphorylation of AKT, BRAF, MEK1/2 and ERK1/2 in xenografts. These are the first data to demonstrate that propranolol might inhibit melanoma by activating the intrinsic apoptosis pathway and inactivating the MAPK and AKT pathways.

## INTRODUCTION

Melanoma, a cancer most commonly affecting the skin, is derived from pigment-containing cells known as melanocytes [1]. In China, the most common subtype is acral lentiginous melanoma, which accounts for approximately 40% of cutaneous melanoma [2, 3]. Surgery followed by systemic chemotherapy is recommended by National Comprehensive Cancer Network (NCCN) for early stage melanoma patients. For unresectable melanoma, BRAF/MEK inhibitors and immune check points inhibitors are the first line treatment [4]. BRAF inhibitors, vemurafenib and dabrafenib, have been approved for use in melanoma patients who are BRAF V600E/V600K mutation carriers, these agents have been shown to significantly improve survival in BRAF-mutated melanoma patients [5, 6]. The MEK inhibitors trametinib can also improve survival in melanoma when used in combination with BRAF inhibitors [7]. However, the duration of response for single-agent vemurafenib varied between 2 to 18 months [8]. Most patients developed an acquired resistance to vemurafenib after 6 months of treatment [9]. Lack of durable response and the development of acquired drug resistance are limitations to the use of BRAF inhibitors, particularly as single-agents.

Mitogen-activated protein kinase (MAPK) and AKT pathways play an important role in BRAF inhibitor antitumor activity. Thep42/p44 MAPK and AKT are highly activated during the growth stage of melanoma [10, 11]. Ras ultimately activates several signaling pathways that are related to cell proliferation and anti-apoptotic signalling cascades, including the Raf/MEK/extracellular signal-regulated kinase (ERK) and phosphatidylinositol 3-kinase (PI3K)/ ATP-dependent tyrosine kinases (AKT)/Phosphatase and PTEN pathways and NF-κB [12, 13]. β-adrenoceptors are also expressed in melanoma cells [14, 15]. Because of the important role of β-adrenergic receptor in regulating AKT/MAPK pathway, we hypothesized that blockage of β-adrenergic receptor may inhibit AKT/MAPK pathway and lead to the death of melanoma cells.

Propranolol is a non-selective β-blocker widely used for the treatment of hypertension, which has also been demonstrated safety and efficacy for the treatment of large hemangiomas in infants [16, 17]. Propranolol has anti-proliferative, anti-migratory, anti-angiogenic and cytotoxic properties in a wide variety of cancers [18–26]. In addition, a retrospective study revealed that melanoma patients received β-blockers for hypertension achieved longer survival than patients did not [27]. Recently, Wrobel*et al.* reported that propranolol, not metoprolol, inhibited proliferation of melanoma cell lines *in vitro* and shrank the tumor size *in vivo*[15]. However, the underlying mechanism of how β-blockers inhibit melanoma remains unknown, especially with the acral melanoma subtype.

This study ascertained if propranolol could inhibit proliferation and induce apoptosis in cutaneous/acral melanoma and explored the potential mechanism of this effect.

## RESULTS

### Propranolol cytotoxicity in cutaneous/acral melanoma

**Figure 1:**
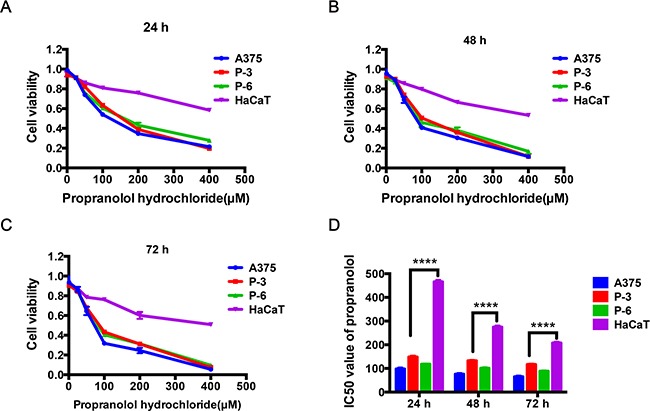
The effect of propranolol on cell survival in melanoma cell lines and normal skin cell line **A-C.** Cell viability was determined following treatment with increasing propranolol concentration (25, 50, 100, 200, 400μM) for 24h, 48h and 72h by AlamarBlue^®^ Cell Viability Assay, relative growth was calculated as the ratio of treated to untreated cells at each dose for each replicate. **D.** IC50 of propranolol after a 24h, 48h and 72h incubation in the four cell lines, respectively. ^****^P<0.0001. All experiments were repeated for at least three times independently.

The direct effect of propranolol on melanoma proliferation was assessed using A375 cell, two acral melanoma cell lines (P-3 and P-6 cell lines), and HaCaT, a normal skin keratinocytes cell line. Cell viability was measured by AlamarBlue^®^ Cell Viability Assay. After treatment for 24h, 48h and 72h with 25μM, 50μM, 100μM, 200μM and 400μM, propranolol respectively, cell proliferation was significantly reduced from 0.911 to 0.056 (Figure 1A, 1B, 1C, *P*<0.0001) in A375, 0.906 to 0.074 (Figure 1A, 1B, 1C, *P*<0.0001) in P-3, and 0.912 to 0.096 (Figure 1A, 1B, 1C, *P*<0.0001) in P-6, respectively. In this assay, propranolol significantly reduced cell viability of the three melanoma cell lines (the range of IC50 in A375: 65.33μM to 98.17, P-3: 116.86μM to 148.60μM, P-6: 88.24μM to 118.23μM, Figure 1D, *P* < 0.05) in a concentration and time dependent manner. Interestingly, compared with melanoma cell lines, propranolol slightly inhibited proliferation of HaCaT cell (the range of IC50 in HaCaT: 88.24μM to 466.11μM, Figure 1D) at the same concentration (less than 200μM).

### Propranolol induced apoptosis and inhibited cell cycle in melanoma cell lines

**Figure 2:**
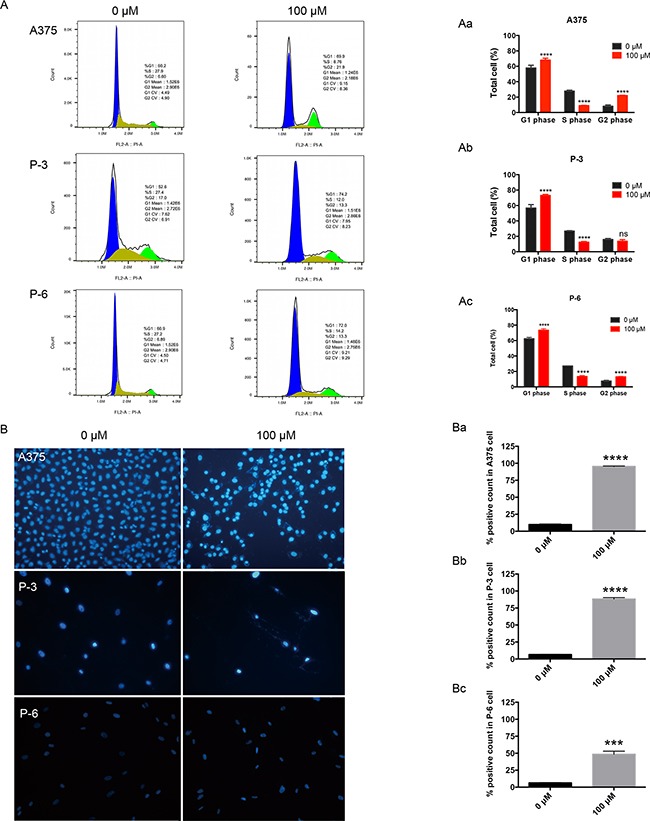
Propranolol induced cell cycle arrest and apoptosis in melanoma cells **A.** A375, P-3 and P-6 cells were exposed to100μM propranolol for 24h and then stained by propidium iodide (PI) to determine cell cycle assay by flow cytometry. **B.** Hoechst staining was performed on the three cell lines after 100μM propranolol treatment. **Aa-Ac**, Data from (A) in histogram. **Ba-Bc**, Quantification of Hoechst staining. ^***^P<0.001, ^****^P<0.0001. All experiments were repeated for at least three times independently.

After 100μM propranolol treatment for 24h, the population of cells in G0/G1 greatly increased from 57.8%, 56.8% and 62.5% to 68.1%, 73.1% and 73.5% in A375, P-3 and P-6 respectively (Figure 2A, Aa-Ac, *P*< 0.0001), the distribution of S phase obviously reduced from 27.9% to 9.0% in A375, 26.8% to 12.5% in P-3 and 27.1% to 13.5% in P-6 (Figure 2A, Aa-Ac, *P*< 0.0001), the number of G2M phase also significantly increased from 8.2% to 22.1% in A375, 7.6% to 13.1% in P-6 (Figure 2A, Aa-Ac, *P*< 0.001), but no significant change in P-3 (Figure 2Ab). This redistribution of cells within the cell cycle may be, at least in part, accounted for propranolol-mediated apoptosis. Hoechst stain showed the apoptotic cell number, defining with chromatin condensation, nuclear shrinkage and formation of apoptotic bodies, significantly increased from 9.9% to 95.4% in A375 (Figure 2B, 2Ba, *P*< 0.0001), 6.6% to 88.3% in P-3 (Figure 2B, 2Bb, *P*< 0.0001), and 6.4% to 48.5% in P-6 (Figure 2B, 2Bc, *P*< 0.0001). These findings suggested that the propranolol might inhibit melanoma cell lines by arresting cell progression at G0/G1 and S phase and then inducing apoptosis.

### Propranolol induced apoptosis by activating an intrinsic apoptosis pathway

**Figure 3:**
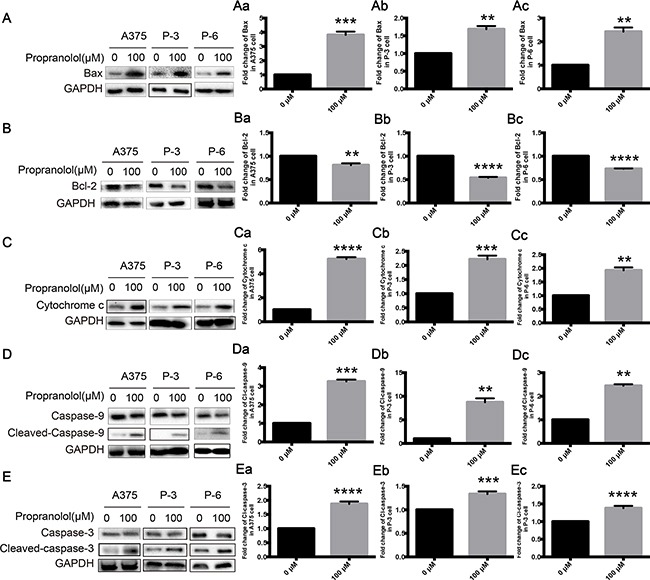
Propranolol activated mitochondria-mediated apoptosis pathway in melanoma **A-E.** The expressions of Bax, Bcl-2, cytochrome c, caspase-9 and caspase-3 following exposure to propranolol 100μM for 24h in A375, P-3 and P-6 cell lines. **Aa-Ec.** Quantification of A-D. Results are presented as mean±SEM, ^*^P<0.05, ^***^P<0.001, ^****^P<0.0001. All experiments were repeated for at least three times independently.

An influence of propranolol on apoptosis may contribute to the mechanistic effects. Melanoma cell lines were exposed to 100μM propranolol for 24h, Bax was significantly increased in A375 (Figure 3A, Aa, *P* <0.001), P-3 (Figure 3A, Ab, *P* <0.01) and P-6 (Figure 3A, Ac, *P* <0.01) cell lines, while Bcl-2 was decreased in the three melanoma cell lines (Figure 3B, Ba-Bc, *P* <0.01). Propranolol also caused releasing of cytochrome c in three cell lines (Figure 3C, Ca-Cc, *P* <0.01). Capase-9 and caspase-3, initiator caspases of the intrinsic apoptotic pathway and one of the downstream target proteins of cytochrome c, were activated by propranolol. An obvious increase of the cleavage of caspase-9 and caspase-3 was observed in A375 (Figure 3D, Da, Figure 3E, Ea, *P* <0.001), P-3 (Figure 3D, Db, Figure 3E, Eb, *P* <0.01) and P-6 (Figure 3D, Dc, Figure 3E, Ec, *P* <0.01) cell lines. These data strongly suggested that propranolol induced apoptosis of melanoma cells by activating intrinsic apoptosis pathway.

### Propranolol inhibited proliferation by inhibiting MAPK pathway

**Figure 4:**
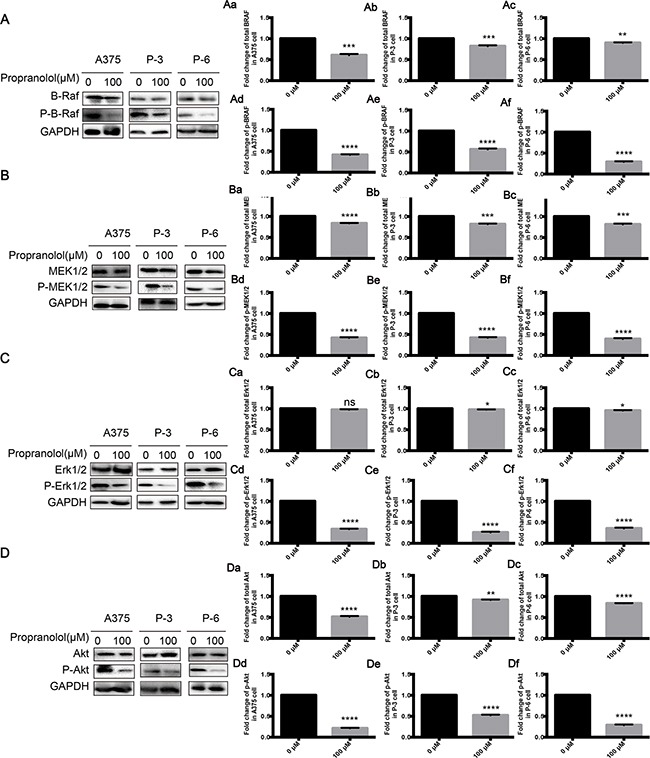
Propranolol inhibited MAPK pathway **A-D.** BRAF, MEK1/2, ERK1/2 and AKT phosphorylation following treatment with propranolol 100μM for 24h in A375, P-3 and P-6 cell lines. Aa-Df, Quantification of A-D. Results are presented as mean±SEM, ^*^P<0.05, ^**^P<0.01,^***^P<0.001, ^****^P<0.0001. All experiments were repeated for at least three times independently.

Since AKT and MAPK pathways play a key role in tumor proliferation and apoptosis, we hypothesized that propranolol might produce its strong function through down-regulating activities in the two pathways. As shown in Figure 4, p-BRAF (Figure 4A, Aa-Ac, *P* < 0.001), p-MEK1/2 (Figure 4B, Ba-Bc, *P* < 0.001), p-Erk1/2 (Figure 4C, Ca-Cc, *P* < 0.001) and p-AKT (Figure 4D, Da-Dc, *P* < 0.001) were greatly reduced while total expressions of them were slightly decreased in propranolol 100μM group after a 24-hour incubation, suggesting that the anti-proliferation might be mediated by simultaneously targeting both ERK and AKT phosphorylation.

### Propranolol inhibited melanoma growth in vivo

**Figure 5:**
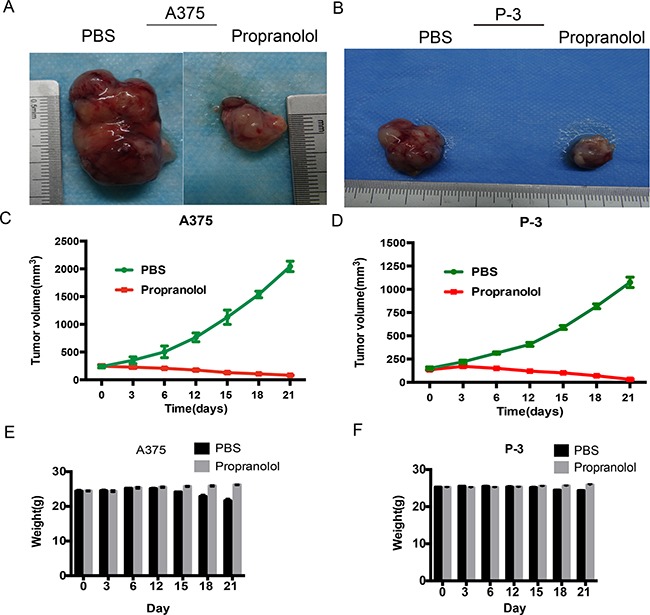
Propranolol inhibits tumor development in xenografts **A, B.** Tumors excised from xenografts of A375 and P-3 model mice, untreated mice (PBS group) and mice treated with propranolol (propranolol group). **C, D.** The growth curves of tumor measured by average volume of 6 tumors in each group. **E, F.** Body weights of A375 and P-3 model mice were measured before and after the beginning of the treatment by three days interval.

To evaluate whether the strong action observed against melanoma *in vitro* could be transferred to xenografts, the three human melanoma cells were engrafted in BALB/C nude mice, while P-6 xenografts were failed to develop to a solid tumor, we compared the effect of propranolol on tumor growth. Previous studies showed propranolol was effective in suppressing melanoma tumor growth in vivo at dosages of 2-10 mg/kg/day [15, 24]. In this study, mice received a daily ip. injection of propranolol at the dose of 2 or 10mg/kg for 3 weeks, the control group was treated with the same volume of saline, but 80% animals were weight loss and hemafecal after the higher dosage propranolol administration (Data not shown), interestingly, the lower showed better effect. As shown in Figure 5, the mean tumor sizes of propranolol-treated mice were smaller than the PBS group on day 21 in A375 xenografts (82.33 ±3.75mm^3^ versus 2044.67 ± 54.57mm^3^, unpaired t-test, *P*<0.0001, Figure 5A, 5C) and P-3 xenografts (31.66 ±4.67 mm^3^ versus 1074.67 ± 32.17 mm^3^, unpaired t-test *P*<0.0001, Figure 5B, 5D). Notably, no mice in the propranolol group lost body weight (more than 10%) both in A375 and P-3 xenografts (Figure 5E, 5F), indicating the absence of gross toxicity for the treatment.

### Propranolol inhibited proliferation and induced apoptosis in melanoma xenografts

**Figure 6:**
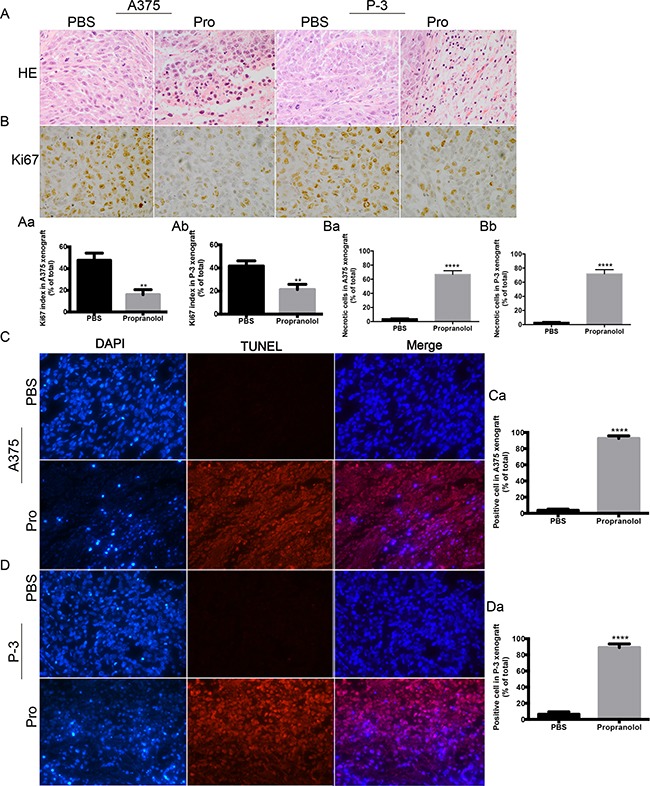
The effect of propranolol on tumor histology *in vivo* **A.** Hematoxylin- -eosin staining (HE) for cell morphology in propranolol and PBS group. **B.** Ki67 was assessed by immunohistochemistry assay. **C-D.** Cell death was measured by TUNEL assay in A375 and P-3 xenografts from untreated (left panels) and propranolol treated mice (right panels). **Ba,-Bb**, Quantification of Ki67 staining in A375 (n=30 estimations on 6 tumors, both in propranolol and PBS groups) and P-3 (n=30 estimations on 6 tumors, both in propranolol treated and PBS groups) xenografts mice. **Ca, Da**, Quantification of TUNEL staining in A375 (n=36 estimations on 6 tumors, both in propranolol and PBS groups) and P-3 (n=36 estimations on 6 tumors, both in propranolol and PBS groups) xenografts mice. Results are presented as mean±SEM, ^**^P<0.01, ^****^P<0.0001. All experiments were repeated for at least three times independently.

To determine whether the inducing apoptosis by propranolol observed *in vitro* also functioned *in vivo*, we performed IHC analyses on tumor sections from all the experimental groups. H&E staining assay was applied to investigate the role of propranolol on cell morphology (Figure 6A). When compared with PBS treated group, the number of nuclei with karyorhexis and karyolysis was greatly increased in propranolol treated xenografts, implying that propranolol could promote cell necrosis in tumors (Figure 6A, Aa, Ab, *P* <0.01). We also assessed the proliferation level indicated by cell marker Ki-67 in tumor sections. The Ki67 index significantly decreased in both A375 (47.67% ± 3.84 % versus 16.00% ± 2.65%, N=6, P= 0.0025, Figure 6B, Ba) and P-3 (41.67% ± 2.60% versus 21.33% ± 2.60%, N=6, P=0.0052, Figure 6B, Bb) xenografts. To demonstrate the role of propranolol on cell death, tunnel terminal deoxynucleotidyl transferase dUTP nick end labeling (TUNEL) assay was performed. In A375 derived tumors, TUNEL positive cell was significantly (*P* < 0.0001) higher of propranolol group (92.67% ± 1.86%) than PBS group (3.67% ± 0.88%, Figure 6C, Ca). In P-3 derived tumors, higher proportion of TUNEL positive cells was observed in the propranolol treated group than in the control group (89.0% ± 2.65% *vs* 6.33% ± 1.76%, *P*<0.0001, N=6, Figure 6D, Da).

### Propranolol inhibited AKT pathway and MAPK pathway in vivo

**Figure 7:**
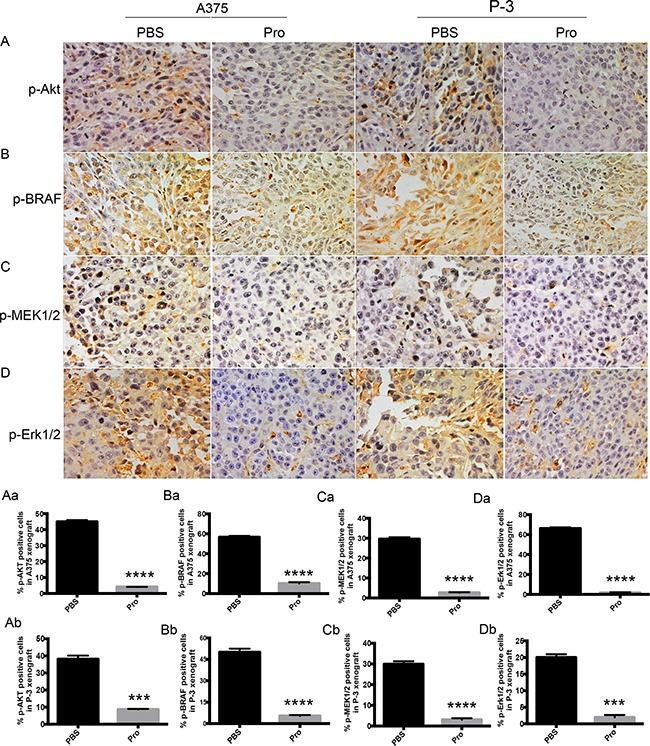
The effect of propranolol on Akt and MAPK pathway *in vivo* **A-D.** p-Akt, p-BARF, p-MEK, and p-ERK were assessed by immunohistochemistry assay in A375 (n=30 estimations on 6 tumors, both in propranolol and PBS groups) and P-3 (n=40 estimations on 6 tumors, both in propranolol and PBS groups) derived xenografts. **Aa-Db.** Quantification of p-AKT, p-BRAF, p-MEK1/2 and p-ERK1/2 staining in A375 (n=30 estimations on 6 tumors, both in propranolol and PBS groups) and P-3 (n=30 estimations on 6 tumors, both in propranolol treated and PBS groups) xenografts mice, respectively. Results are presented as mean±SEM, ^***^P<0.001, ^****^P<0.0001. All experiments were repeated for at least three times independently.

To determine whether the down-regulation of MAPK and AKT signaling by propranolol observed *in vitro* also functioned *in vivo*, immunohistochemistry analysis was applied to determine the phosphorylation of the AKT and MAPK pathways in tumors. As observed in Figure 7, the data showed that the expression of p-AKT was significantly (P<0.0001) reduced in the propranolol treated group compared with PBS group both in A375 (Figure 7A, Aa) and P-3 xenografts (Figure 7A, Ab). The expression of p-BRAF and p-MEK1/2 were moderately lowered by propranolol in A375 (Figure 7B, 7C, Ba, Ca) and P-3 xenografts (Figure 7B, 7C, Bb, Cb). Furthermore, the phosphorylated ERK1/2 was reduced in A375 (Figure 7D, Da) and P-3 (Figure 7D, Db) derived tumors in the propranolol treated group. These data revealed that propranolol could inhibit the growth of melanoma by inhibiting the phosphorylation of AKT and MAPK pathway *in vivo*.

## DISCUSSION

Accumulative studies have reported that β-blockers have anti-tumor effects by inhibiting proliferation, inducing apoptosis and inhibiting metastasis of many types of tumor [24–26]. Calvani M and colleagues found β3-AR activation in melanoma accessory cells drives stromal reactivity by inducing pro-inflammatory cytokines secretion and de novo angiogenesis, sustaining tumor growth and melanoma aggressiveness, which validated selective β3-AR antagonists as potential promising anti-metastatic agents [28]. Another study demonstrated the combination of propranolol with 5-FU or paclitaxel resulted in more profound and sustained anti-tumor effects and significantly increased the survival benefits in MDA-MB-231 cell xenografts [20]. In 2015, Wrobel*et al*. reported that propranolol could inhibit proliferation in melanoma cell lines without knowing its underlying mechanism [15]. This study demonstrated that propranolol inhibited melanoma growth by regulating the AKT pathway and MAPK pathways and inducing apoptosis. It is also the first evidence showing that activation of apoptosis by propranolol could down-regulate the activity of AKT and MAPK pathways both in cutaneous and acral melanoma.

Propranolol inhibited proliferation in the three melanoma cell lines. Wrobel and his colleague also addressed that propranolol significantly reduced melanoma cell viability with 100μM for 24h [15]. Similar result was reported by Albiñana who found that propranolol reduced viability and induced apoptosis in hemangioblastoma cells from von Hippel-Lindau patients [29]. Consistent with the *in vitro* studies, propranolol shrunk tumor size and inhibited the expression of Ki67 in xenograft models. In neuroblastoma and breast cancer, propranolol was also observed to reduce tumor volume in xenografts models [20, 24]. Propranolol inhibition of proliferation of cutaneous and acral melanoma occurred in a time and concentration dependent manner. This observation is consistent with the hemangioblastoma study, which found 50 and 100μM propranolol significantly reduced cell viability with longer incubation time (24h-96h) [29]. Liao and his colleague also demonstrated that propranolol reduced proliferation of gastric cancer cells in a concentration-dependent manner (25μM-300μM)[23]. Pharmacokinetic data suggested that the peak serum concentrations of propranolol ranged from 200-400 ng/ml, which is equivalent to 0.77-1.5μM *in vivo*[30]. But the IC50 value of propranolol was more than 60 μM after a 24-hour incubation in three melanoma lines. Large discrepancy of drug concentration was observed between *in vivo* and *in vitro* models. This may because propranolol could exert the anti-tumor effect via multiple pathways *in vivo* compared with *in vitro* models, such as propranolol, could influence angiogenesis [31, 32] and attenuate immune-suppressionin tumor [33], thus may require less drug concentrations to achieve similar effect. On the other hand, propranolol showed no effect on normal skin cell line at the same concentration. This is consistent with a recent study, which demonstrated that propranolol did not show any cytotoxicity in normal skin cells and normal melanocytes [15].

The mechanisms of anti-tumor activity of β-blockers have been explored by other investigators in other cancer. Lin X et al. found that the non-selective β-AR agonist isoproterenol significantly increased the activation of ERK/MAPK signal pathway in pancreatic cancer cell [25]. Huang's study showed that norepinephrine also stimulated pancreatic cancer cell proliferation, migration and invasion via β-AR dependent activation of P38/MAPK pathway [34]. Propranolol and other β-blockers reduced the activity of MAPK in pancreatic cancer and Hemangioma [35, 36]. Meanwhile, inhibition of MAPK is a widely used strategy for melanoma treatment [37]. Vemurafenib, a BRAF^V600E^ inhibitor, has achieved high response rate (more than 50%) at the initial stage of vemurafenib treatment in advanced melanoma patients [38–40]. However, more than 60% patients developed acquired drug resistance (ADR) after 6 months [41–43]. One possible reason of vemurafenib induced ADR was believed to be the reactivation of MAPK pathway in melanoma [44]. This study further advanced our knowledge on the therapeutic mechanism of propranolol in melanoma. More significantly, our data also provide a scientific basis on a strategy of using the propranolol inhibitory effects on the MAPK pathway to overcoming the vemurafenib-induced resistance in melanoma treatment.

In summary, this data implied that propranolol could inhibit melanoma *in vitro* and *in vivo*. The study highlights for the first time that propranolol may exert the anti-tumor effect in cutaneous and acral melanoma by suppressing AKT and MAPK pathways. Clinical trials are needed to verify the treatment effect of propranolol in melanoma patients as an adjuvant regimen.

## MATERIALS AND METHODS

### Cell lines and reagents

The A375 cell line, derived from chronic sun-induced damage (CSD) cutaneous melanoma [45], was obtained from American type culture collection (ATCC). All cell lines were cultured in DMEM medium (Gibco, Life Technologies, China) supplemented with 10% FBS (Gibco, Life Technologies Australia) at 37°C and 5% CO_2_ in tissue culture incubator. The A375 cell line was regularly verified to be mycoplasmae –free and no cross cell contaminationusing ABI 3100 type genetic analyzer (Applied Biosystems, USA). (Supplementary Figure S1).

### Human melanoma primary cultures

Patient 3 cell line (P-3) and patient 6 cell line (P-6) were derived from surgical resection samples of acral melanoma from two patients (Supplementary Figure S2). Written informed consent was obtained from the patients. Melanoma specific antigens were detected via western blotting for CD31, CD63, CD166 and CD146 confirmed that the cells were melanocytes (Supplementary Figure S3, Supplementary Figure S6). Tumor tissues were mechanically dissociated with a small tissue chopper prior to sequential enzymatic digestion in 2 mg/ml Collagenase (Sigma-Aldrich, Schnelldorf, Germany) and 1 mg/ml Dispase I (Gibco/Invitrogen, Carlsbad, CA) in DMEM for 30 min at 37°C. Cells were filtered (100 μm cell strainer) to obtain a single cell suspension and re-supplemented by phosphate-buffered saline, thereafter centrifuged at 1000 r.p.m. for 5 minutes. Pellet was re-suspended in DMEM with 10% FBS and then cultured in 75 cm^3^ plastic flasks (Themo Fisher Scientific, China).

### Adenylyl cyclase activity

Adenylyl cyclase activity was measured by a protein binding assay [46]. Cell pellets were thawed and homogenised in a glass douncehomogeniser containing ice-cold homogenization buffer (0.3 M sucrose, 25 mMTris, pH 7.4). A 40 μL sample of homogenate was then added to 30μL premix buffer (final assay concentration 50 mMTris (pH 7.5), 5 mM Mg^2+^, 1 mM ATP (Sigma-Aldrich, Schnelldorf, Germany), 1μM GTP (G9002-25MG,Sigma-Aldrich, Schnelldorf, Germany), 250μM 4- (3-Butoxy- 4-methoxybenzyl) imidazolidin-2-one (RO20-1724, Abcam, USA), 20 mMcreatine phosphate (000000010621714001, Roche) and 130 U/mL creatine phosphokinase (C7886-500UN, Sigma-Aldrich, Schnelldorf, Germany)). The tubes were incubated at 37°C for 10 min and the reaction terminated by the addition of 20 μL of 100% trichloroacetic acid and the tubes placed on ice for 10 min. Precipitated protein was pelleted by centrifugation at 2,900 × g for 20 min at 4°C, the resulting supernatant was used to determine cAmp level by cAMP Direct Immunoassay Kit (Catalog#K371-100, BioVison, USA) according to the instruction. The adenylyl cyclase activity expressed as pmol cyclic AMP min^−1^ mg ^−1^ protein.

### Quantitative real-time PCR (qRT-PCR)

Total RNA was extracted from the three melanoma and HaCaT cell lines by using Trizol reagent (Invitrogen, Carlsbad, CA) according to the manufacturer's instructions. qRT-PCR was performed using the SYBR® Green Realtime PCR Master Mix assay kit (Toyobo, Osaka, Japan) according to the manufacturer's instructions. The primers of target genes were as follows: β1AR: 5′-ATCGAGACCCTGTGTGTCATT-3′ (forward) and 5′-GTAGAAGGAGACTACGGACGAG-3′ (reverse), β2AR: 5′-TTGCTGGCACCCAATAGAAGC-3′ (forward) and 5′-CAGACGCTCGAACTTGGCA-3′ (reverse), β3AR: 5′-GACCAACGTGTTCGTGACTTC-3′ (forward) and 5′-GCACAGGGTTTCGATGCTG-3′ (reverse), and GAPDH: 5′-GAGTCAACGGATTTGGTCGT-3′ (forward) and 5′-TTGATTTTGGAGGGATCTCG-3′ (reverse). The results were analyzed using the 2^−ΔΔCt^ method as the following formula: ΔΔCt = ΔCt_β2/β3AR_-ΔCt_β1AR_, ΔCt = Ct _target gene_ -Ct_GAPDH_.

### Cell viability assays

The half-maximal inhibitory concentration (IC50) value was determined by AlamarBlue^®^ assay (Invitrogen, Burlington, ON, Canada). A375 cells were plated in 96-well plates at a density of 2×10^3^ and treated with 25μM-400μM propranolol (propranolol hydrochloride, P0884, Sigma-Aldrich, USA) for 24h, 48h and 72h. Cells were incubated with 10% of AlamarBlue overnight. Fluorescence of each plate was measured using a spectrophotometer at excitation 530 nm and emission 590 nm (Spectra MAX Gemini EM, Molecular Devices).

### Cell cycle analysis by flow cytometry

A375,P-3 and P-6 cell lines were cultured at a density of 1 × 10^6^ cells in 100 mm^2^ culture dishes (Corning, China) and were treated with 100 spectrophotometer 24h. Cells were then harvested, washed, fixed overnight in 70 % ethanol at −20°C, after washed with PBS, digested with RNase A, stained with propidium iodide (50 with propidiumilyzed by flow cytometry using BD Accuri™C6 (Becton, Dickinson and Company, USA).

### Western blot analysis

Western blot analysis was performed on cell extracts of A375,P-3 and P-6 cell lines treated with 100μM propranolol for 24h. Immunoblots were performed from whole cell lysate prepared using RIPA Buffer (Cell Signaling Technology) supplemented with dithiothreitol (DTT), phenylmethylsulphonyl fluoride (Sigma), and fresh protease and phosphatase inhibitors (Sigma). Cell lysates were quantified for protein content using a bicinchoninic acid (BCA) protein assay kit (Thermo). Protein samples were resolved on NuPAGE 12% Bis-Tris gels with MOPS buffer or 3–8% Tris acetate gels with Tris acetate buffer (Life Technologies) and then transferred to 0.45-mm nitrocellulose membrane (Bio-Rad). After saturation in Tris-buffered saline supplemented with 5% BSA, the membranes were incubated with antibodies (diluted at 1:2,000) overnight at 4°C. Antibodies specific for the following proteins were purchased from Cell Signaling Technology: AKT (rabbit, 9272), B-Raf (rabbit, 9433), MEK1/2 (L38C12) (mouse, 4694), ERK1/2 (rabbit, 9102), phospho-ser473-AKT (rabbit, 9271), phosphor-ser445-BRAF (rabbit, 2696), phosphor-ser221-MEK (rabbit, 2338), phospho-ERK1/2-Thr202/Tyr204 (rabbit, 4370), cytochrome c-D18C7 (rabbit, 11940), caspase-9 (mouse, 9508) and caspase-3 (rabbit, 9662). The antibodies specific for Bax (rabbit, sc-6236) and Bcl-2 (rabbit, sc-492), were purchased from Santa Cruz Biotechnology. The antibodies for BAD (rabbit, ab32445), p-BAD (rabbit, ab28824), CD31 (mouse, ab9498), CD147 (rabbit, ab108317), CD146 (mouse, ab24577), CD63 (mouse, ab8219) and CD166 were purchased from Abcam. The antibody specific for GAPDH (mouse, clone 6C5, MAB374) was purchased from Millipore. Quantification of the bands was done with Image J.

### Hoechst staining

Cells treated with propranolol were fixed, washed twice with PBS and stained with Hoechst 33258 staining solution according to the manufacturer's instructions (Beyotime, Jiangsu, China). Stained nuclei were observed under a fluorescence microscope (Leica-microsystems-DM5000B+DFC1300,Leitz Wild Group, Germany).

### Xenografts of human melanoma

The six weeks old NOD/SCID male mice (Hunan Silaike experimental Animals Inc, China) was injected subcutaneously with 10^6^living melanoma cells that were maintained in DMEM with 2% FBS and 15% Matrigel (BD Bioscience, Franklin Lakes, NJ) in the left flank. A solid tumor develops in A375 and P-3 xenografts within two weeks, while P-6 xenografts were failed to develop.

### In vivo propranolol treatment

The NOD/SCID mice with xenografts were randomly divided into three groups after the tumor volume reached 0.3 cm^3^. The propranolol group (n=6) received a daily ip. injectionof propranolol (Sigma-Aldrich) at the dose of 2 or 10mg/kgfor 3 weeks and the control group (n=6) was treated with the same amount of saline. Tumor volume was measured by length (l), width (w), and height (h) twice a week with an external caliper and tumor size was calculated by the modified ellipsoidal formula (Tumor volume =π/6× l × w × h). The mice were sacrificed at the end of 3 weekstreatment. The xenografts were removed, weighted and then snap-frozen in liquid nitrogen. Paraffin-embedded tumor blocks were prepared for further analysis at the same time.

### Immunohistochemistry and TUNEL assay

Deparaffinized tissue sections were treated with Antigen Retrieval Solution (made from citrate buffer, pH 6.0, concentrated 103, T0050 Diapath). Tissue sections were then incubated with Peroxidase Blocking Solution (S2023, Dako) for 15 min and Protein Block (X0909, Dako) for 20 min. Primary antibody specific for phosphor-ser221-MEK (rabbit, 2338, CST), phospho-ERK1/2-Thr202/Tyr204 (rabbit, 4370, CST) and phospho-ser473-AKT (rabbit, 9271, CST) and p-RAF-B (goat, sc-28006, Santa Cruz Biotechnology) were applied, and the slides were incubated overnight at 4°C. Signals were visualized using rabbit HRP-conjugated secondary antibody (K4003, Dako) and a haematoxylin (MHS32, Sigma) counterstain. TUNEL assay was done using Cell-Light™EdUTP TUNEL In situ Detection Kit (Ruibo, China).

### Statistical procedures

Drug sensitivity of propranolol and cell cycle population analysis were done using an analysis of variance on repeated measures with the Graphpad Prism software (GraphPad Software, Inc., version 6.0).

## SUPPLEMENTARY MATERIALS FIGURES






