# Supplementary material for: Propranolol induced G0/G1/S phase arrest and apoptosis in melanoma cells via AKT/MAPK pathway
Source: Oncotarget. 2016 Aug 25;7(42):68314–27. doi: 10.18632/oncotarget.11599 (PMC5356557; doi:10.18632/oncotarget.11599)
Supplement: Supplementary file 1 [file oncotarget-07-68314-s001.pdf]

Propranolol induced G0/G1/S phase arrest and apoptosis in melanoma cells via AKT/MAPK pathway

SUPPLEMENTARY FIGURES

Supplementary 1: Report of Human Cell Line Authentication.

See Supplementary File 1

Supplementary 2: Pathology report of the two patients.

See Supplementary File 2

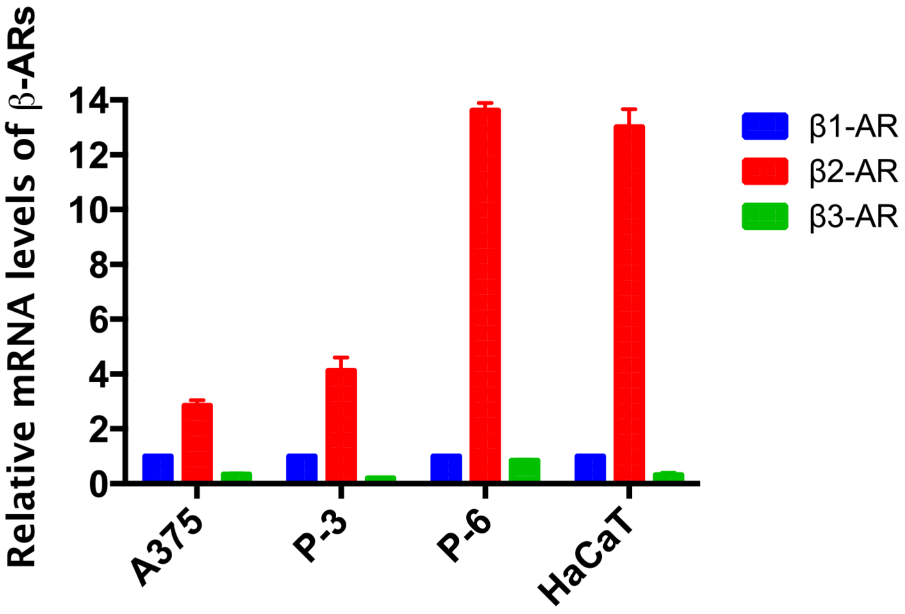

Supplementary Figure S1: Expressions of β-ARs in A375, P-3, P-6 and HaCaT cell lines.

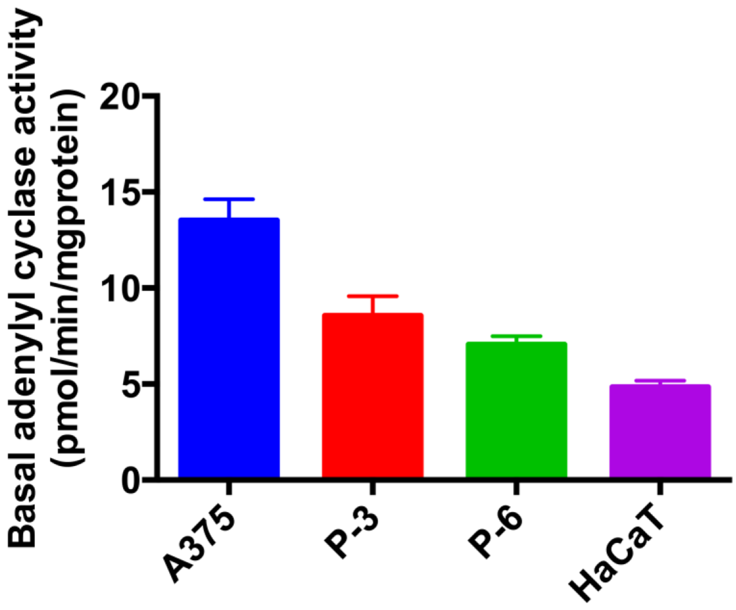

Supplementary Figure S2: The activity of β-ARs in A375, P-3, P-6 and HaCaT cell lines.

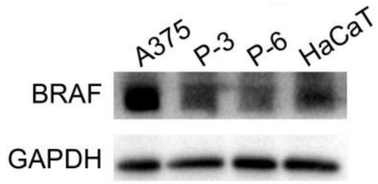

Supplementary Figure S3: The BRAF status in A375, P-3, P-6 and HaCaT cell lines.

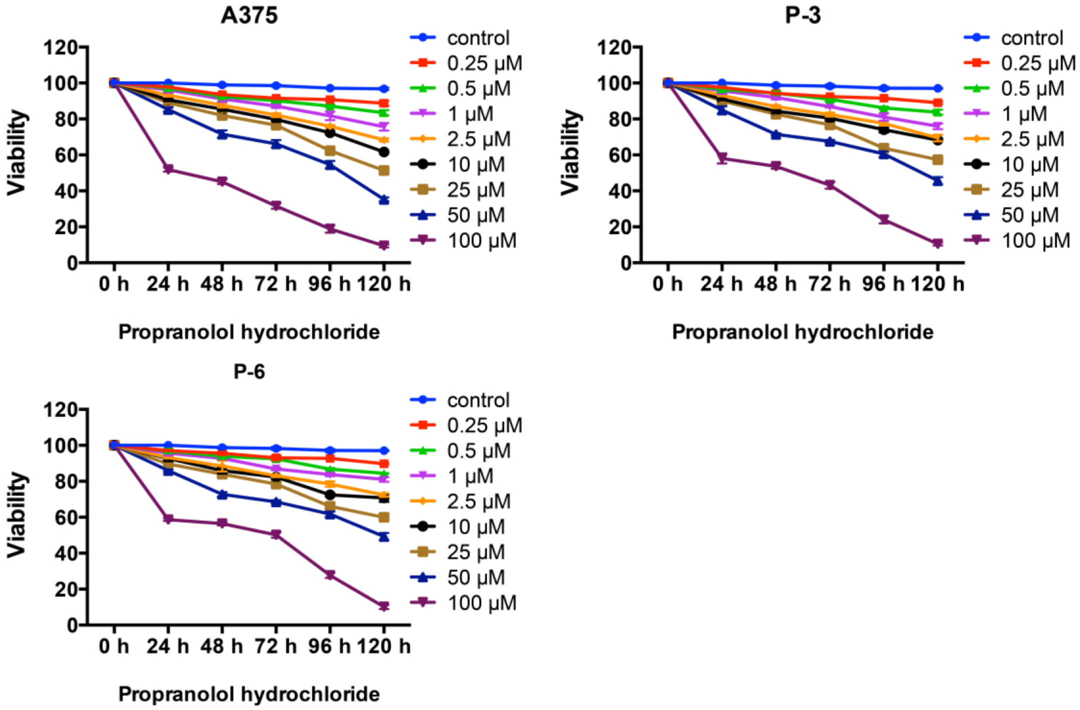

Supplementary Figure S4: 0.25μM-100μM of propranolol reduced cell viability with chronic exposure.

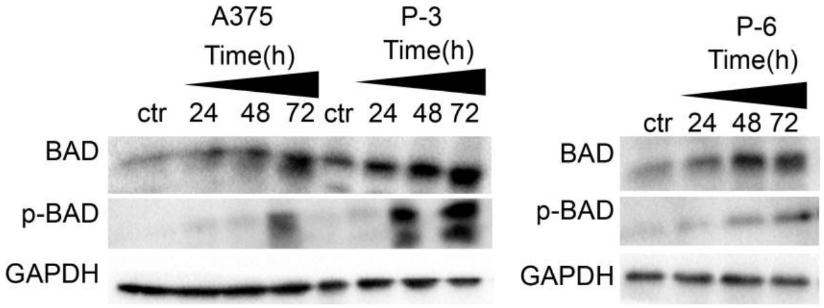

Supplementary Figure S5: 10μM propranolol increased the levels of p-BAD and BAD in a time dependent manner.

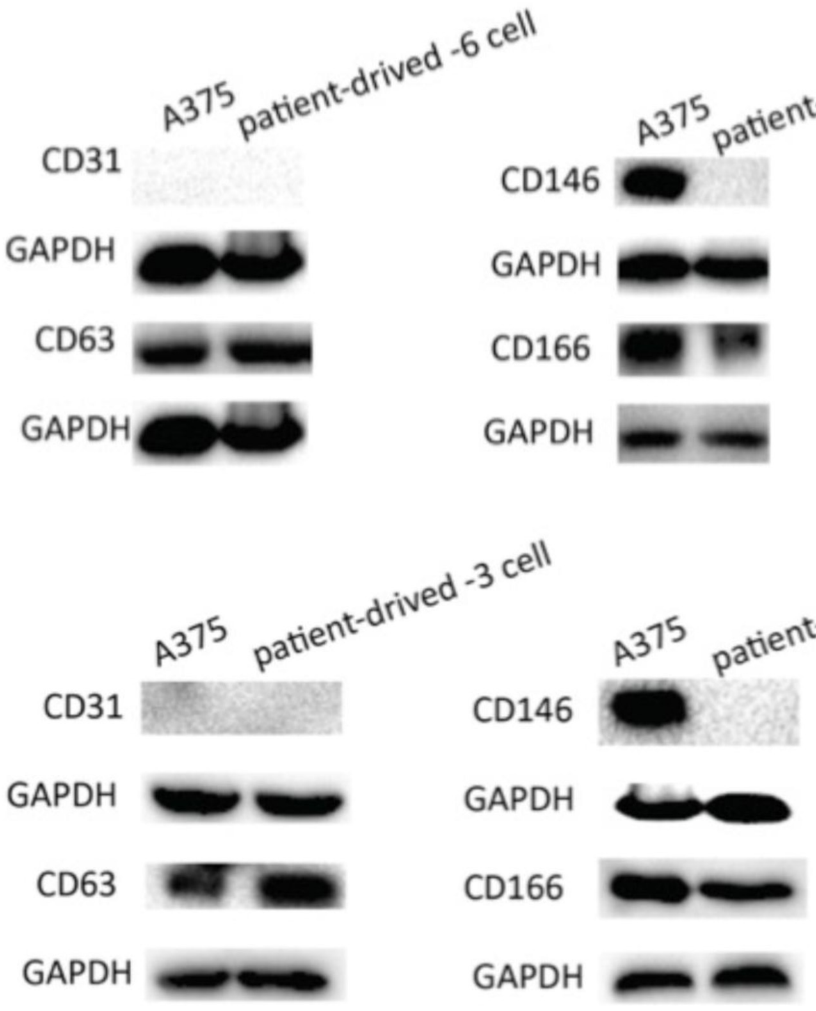

Supplementary Figure S6: P-3 and P-6 melanoma cells were defined as CD45—/CD31— cells co-expressing one or more melanoma-related antigens (CD63, CD146, CD166).
