# Supplementary material for: Propranolol induced G0/G1/S phase arrest and apoptosis in melanoma cells via AKT/MAPK pathway
Source: Oncotarget. 2016 Aug 25;7(42):68314–27. doi: 10.18632/oncotarget.11599 (PMC5356557; doi:10.18632/oncotarget.11599)

**Report of Human Cell Line Authentication**

Delivery Date: February 28^th^, 2015

Analysis Date: March 5^th^, 2015

Ⅰ. Sample

Sample Name: ‘XB2800’, [labeled](http://dict.cnki.net/dict_result.aspx?searchword=标记&tjType=sentence&style=&t=labeled) as ‘VCF1’, and was received on February 28^th^, 2015.

Ⅱ. Method and Procedure

1. PCR is amplified with STR Multi-amplification Kit (Microreader^TM^21 ID System);

2. PCR products are assayed with 3100 DNA Analyzer (Applied Biosystems®).

Ⅲ. Results

1. The results of the negative and positive control match expectations.

2. The STR profiles of the cell line sample are in the attached table and figure.

VCF1: ①No cross-contamination of other human cell line is found. ②100% matched cell lines are found in ATCC and DSMZ data banks. And the cell line named as A-375 *et al*. No 100% matched cell line is found in the [JCRB](http://cellbank.nibio.go.jp/cellbank_e.html) data bank.

Operator: Mengjuan He Auditor: Chen Li

Beijing Microread Genetics Co.,Ltd

Table: STR profiles of VCF1 cell line

| Cell line VCF1（Fig. XB2800） | | |
| --- | --- | --- |
| Marker | Allele1 | Allele 2 |
| D19S433 | 13 | 14.2 |
| D5S818 | 12 | 12 |
| D21S11 | 29 | 30 |
| D18S51 | 12 | 17 |
| D6S1043 | 11 | 14 |
| AMEL | X | X |
| D3S1358 | 15 | 17 |
| D13S317 | 11 | 14 |
| D7S820 | 9 | 9 |
| D16S539 | 9 | 9 |
| CSF1PO | 11 | 12 |
| Penta D | 9 | 15 |
| D2S441 | 11 | 11 |
| vWA | 16 | 17 |
| D8S1179 | 11 | 14 |
| TPOX | 8 | 10 |
| Penta E | 10 | 12 |
| TH01 | 8 | 8 |
| D12S391 | 18 | 21 |
| D2S1338 | 16 | 24 |
| FGA | 23 | 23 |

Figure: STR profiles of VCF1 cell line


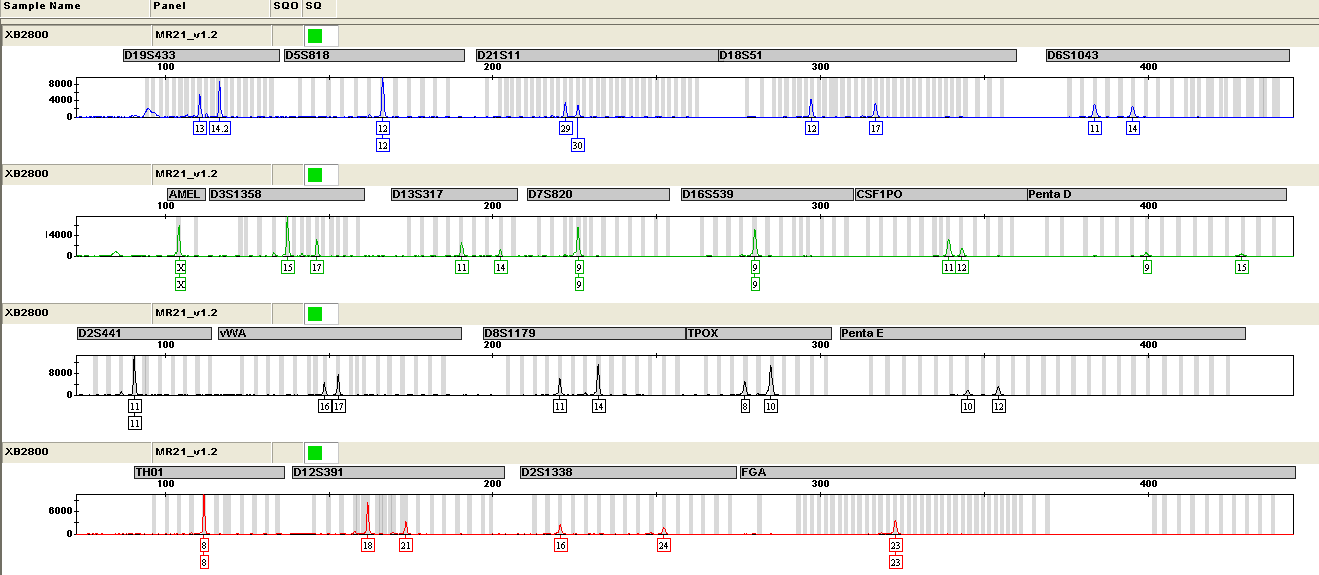


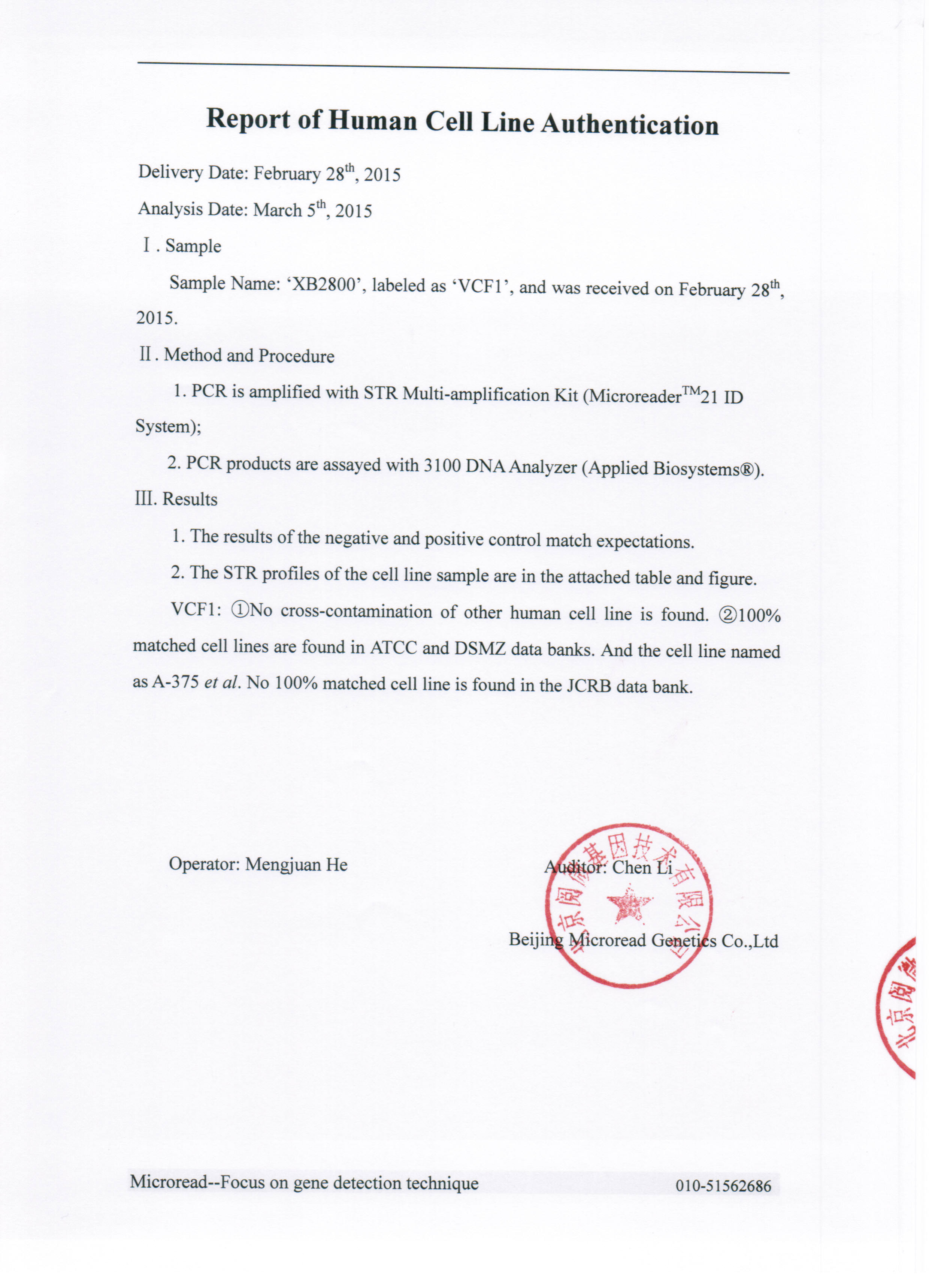


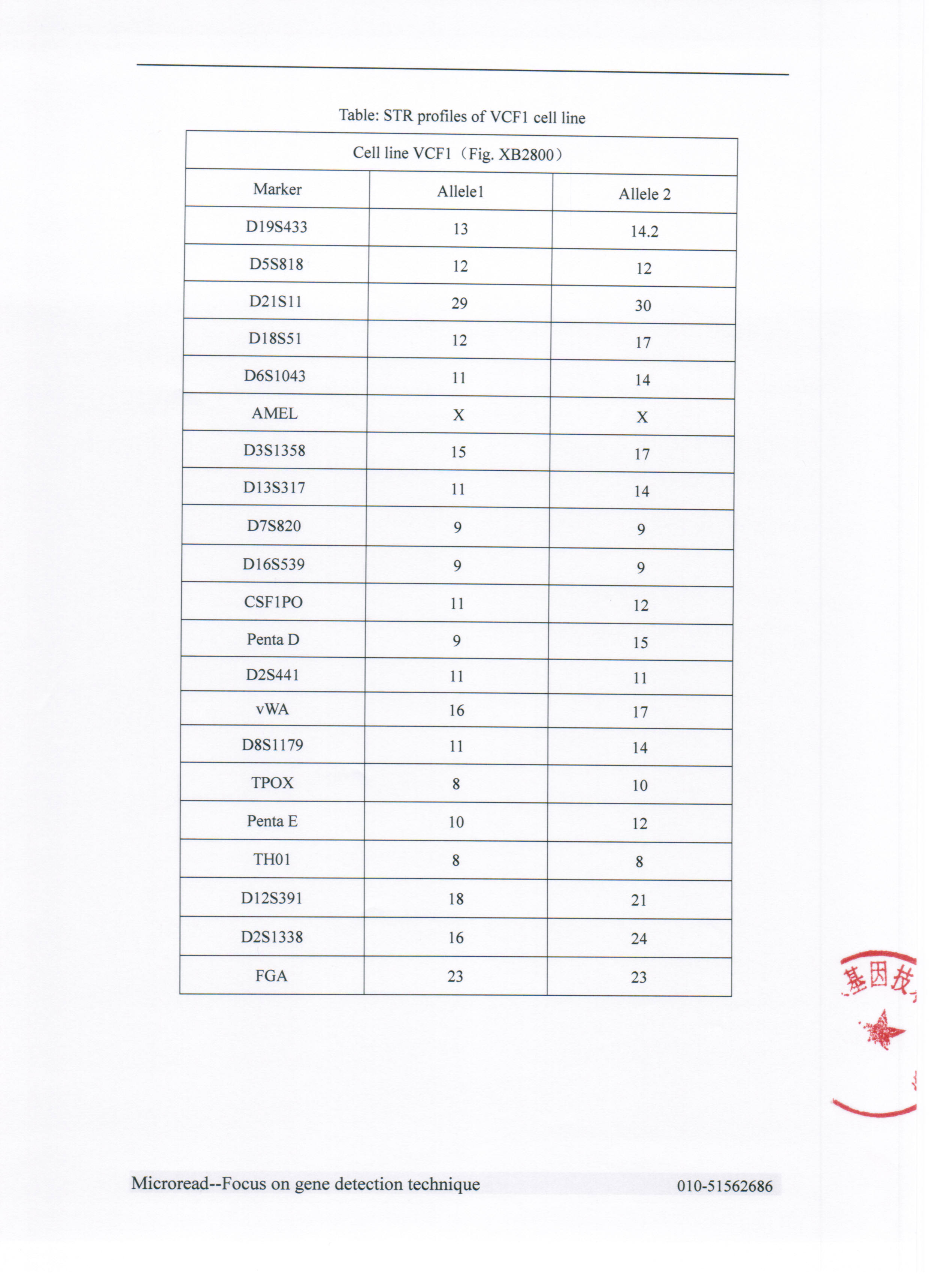


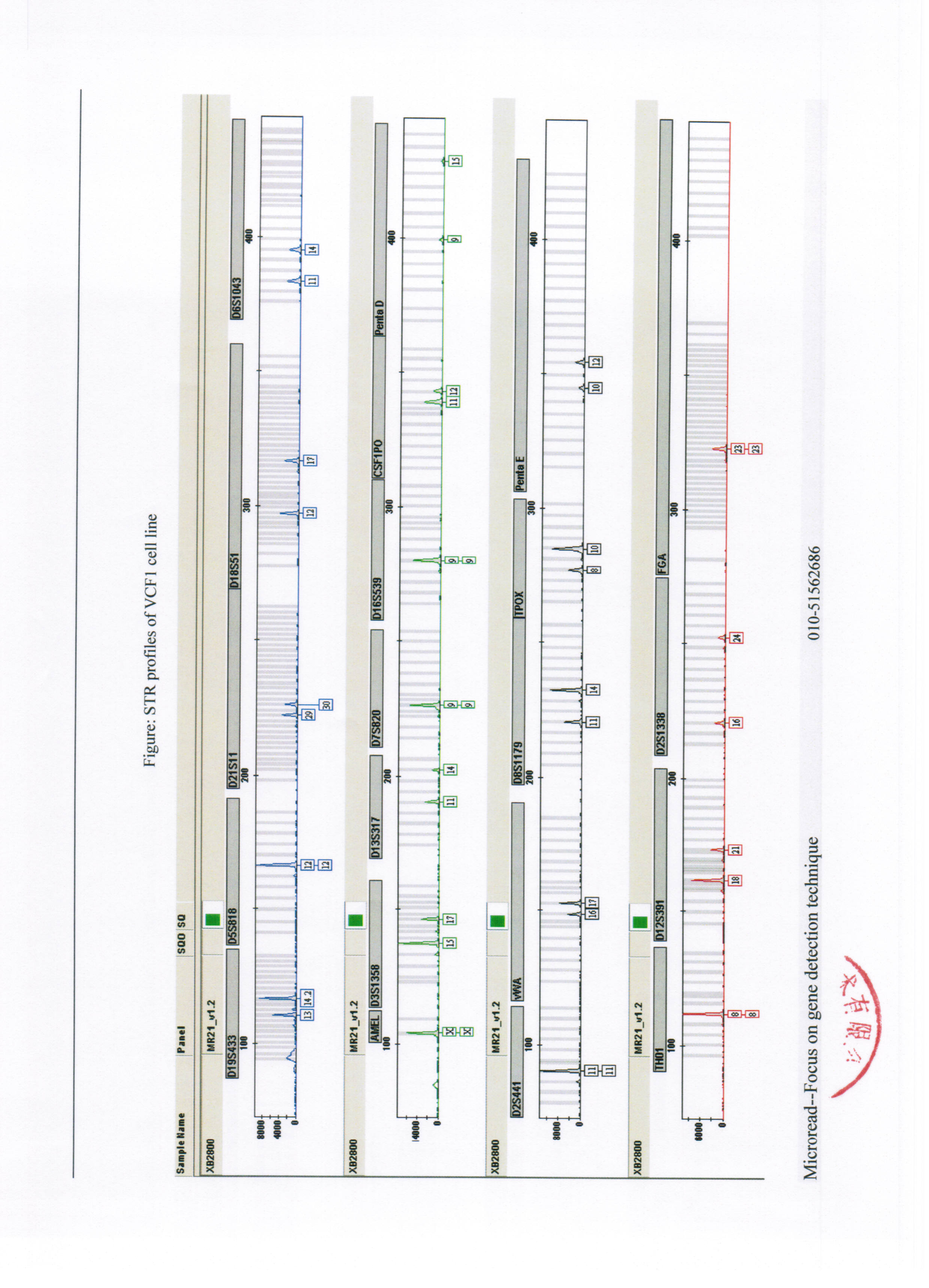

Supplement: Supplementary file 2 [file oncotarget-07-68314-s002.doc]
