# Supplementary material for: Propranolol induced G0/G1/S phase arrest and apoptosis in melanoma cells via AKT/MAPK pathway
Source: Oncotarget. 2016 Aug 25;7(42):68314–27. doi: 10.18632/oncotarget.11599 (PMC5356557; doi:10.18632/oncotarget.11599)
Supplement: Supplementary file 3 [file oncotarget-07-68314-s003.docx]

**Pathology report of the two patients**

1. Pathology report of patient 3(derived P-3 cell line).


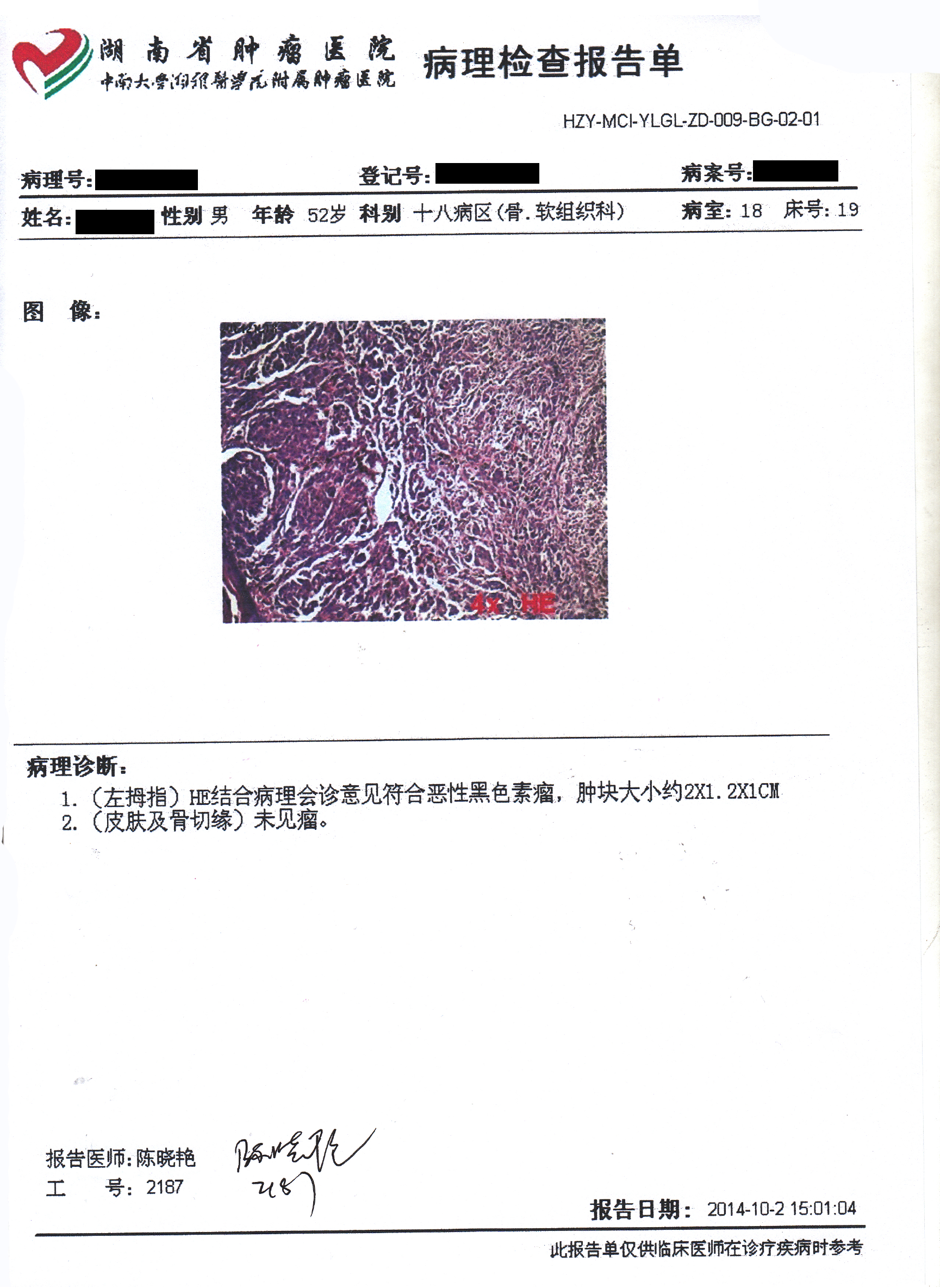


| **Pathology Report**  (Hunan Tumor Hospital) |
| --- |
| Path. No.: XXX Reg. No.: XXX Patient ID No.: XXX |
| Name: XXX Sex: male Age: 52 Ward No.: 18 Hospital Bed No.: 19  Department: Bone and Soft Tissue Sarcomas |
| Image: |
| DIAGNOSIS:   1. Melanoma, acral type, left thumb, biopsy, tumor size: 2×1.2×1cm. 2. No invasion in deep soft tissue and bone.   Pathologist: Xiaoyan Chen  Pathologist ID No.：2187  Date: Oct.2nd, 2014 |

1. Pathology report of patient 6(derived P-6 cell line).


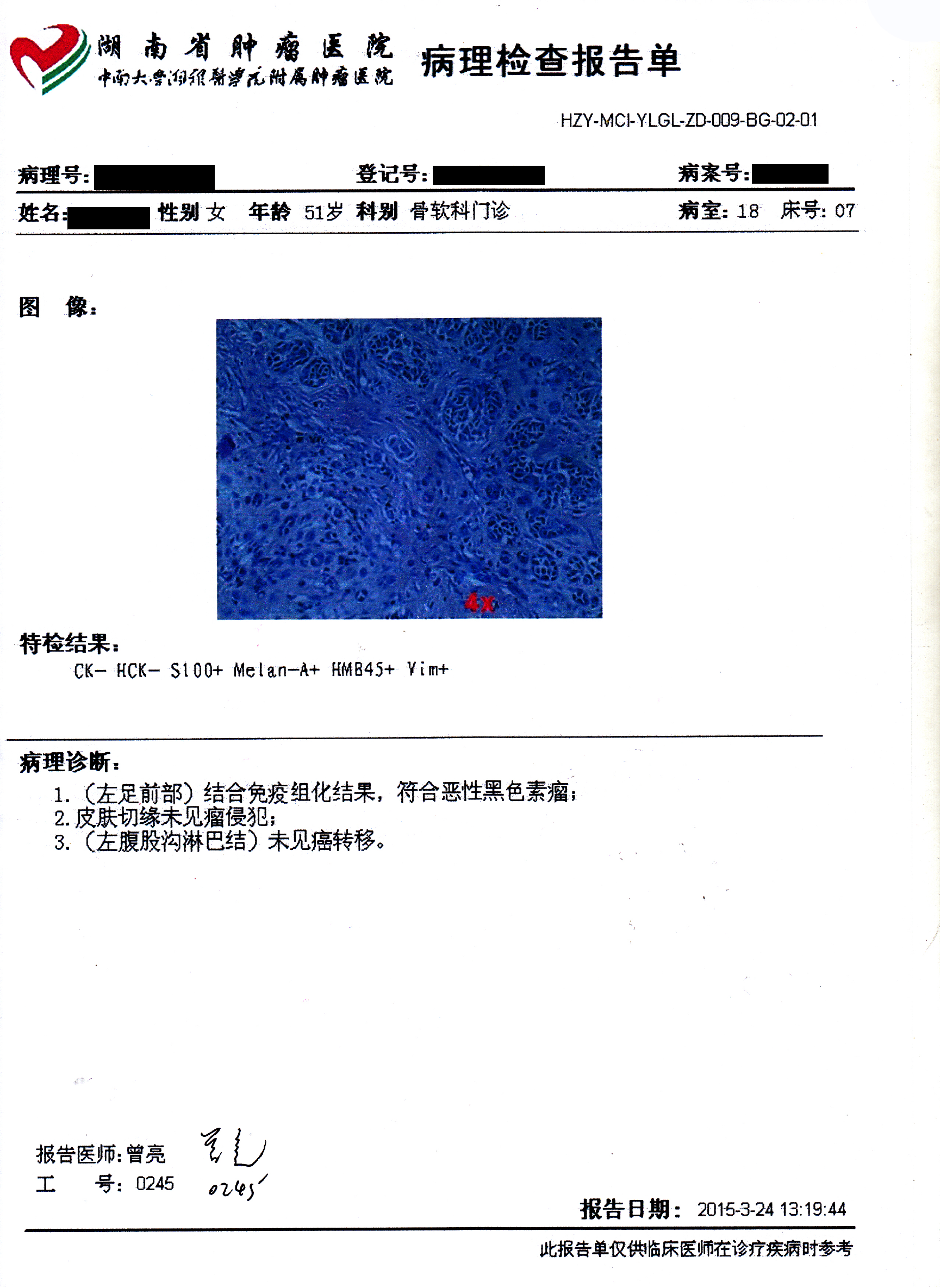


| **Pathology Report**  (Hunan Tumor Hospital) |
| --- |
| Path. No.: XXX Reg. No.: XXX Patient ID No.: XXX |
| Name: XXX Sex: Female Age: 51 Ward No.: 18 Hospital Bed No.: 07 Department: Bone and Soft Tissue Sarcomas |
| Image:  Specific immunohistochemistry: CK－,HCK—, S100+ ,Melan-A+, HMB45+,Vim+, |
| DIAGNOSIS:   1. Melanoma, acral type, front of the left foot, biopsy 2. No invasion in skin incisal margins 3. Left inguinal lymph nodes involvement not identified   Pathologist: Liang Zeng  Pathologist ID No.：0245  Date: Mar.24th, 2015 |
